# Staff and service user perspectives of a co-located homelessness centre in Scotland: a mixed-methods evaluation

**DOI:** 10.3399/BJGPO.2024.0198

**Published:** 2025-07-02

**Authors:** Lauren Ng, Eddie Donaghy, John Conway, Stewart W Mercer

**Affiliations:** 1 Usher Institute, College of Medicine and Veterinary Medicine, University of Edinburgh, Edinburgh, UK; 2 Cyrenians, Edinburgh, UK

**Keywords:** homelessness, integration, Scotland, survey

## Abstract

**Background:**

Co-location and integration of services within a psychologically informed environment (PIE) is recommended for people experiencing homelessness (PEH) but there are few examples of this in the UK. Such a centre opened in Edinburgh, Scotland in November 2021.

**Aim:**

To evaluate progress of the new centre.

**Design & setting:**

This was a mixed-methods pre–post-test design study before (baseline) and 2 years after (follow-up) the move to the new co-located centre. The study took place in Edinburgh, Scotland. The baseline evaluation was conducted at two separate homelessness services and the follow-up evaluation at the new co-located centre.

**Method:**

Baseline and follow-up staff surveys measured knowledge of trauma-informed care (TIC), wellbeing, team climate, and job satisfaction. The follow-up staff survey also evaluated staff support and service improvements. In-depth staff interviews were conducted at baseline (*n* = 25) and follow-up and analysed thematically. A service-user survey was also conducted.

**Results:**

The staff survey showed significant improvements between baseline and follow-up in TIC, burnout, and team climate, together with improvements in support, service integration, and service-user care. Service users reported high satisfaction with the new centre. Staff interviews identified a more PIE, better staff support, and improved opportunistic multidisciplinary working over the 2 years of the centre opening. However, a number of barriers were also identified relating to the building and the IT systems. Further work on the centre’s vision, short and long-term integration plans, workload, and sustainability were felt to be needed.

**Conclusion:**

Co-location of services for PEH in Scotland has led to reductions in staff burnout and improvements in team climate and service users’ satisfaction over the first 2 years of opening. However, barriers remain and full integration requires a clearer vision and ‘roadmap’, requiring collaborative leadership and sustainable funding.

## How this fits in

In keeping with the National Institute for Health and Care Excellence’s (NICE’s) guidance on care integration for people experiencing homelessness (PEH), Edinburgh’s homelessness services co-located into one building under a single-line management structure in 2021, with a psychologically informed environment (PIE) underpinning the centre to better support PEH and staff. This evaluation spanned the move from the previous separate homelessness services (situated in different areas in Edinburgh) to the new co-located centre; and to the best of our knowledge, this is the first study evaluating a co-located homelessness centre in the UK and wider Europe. Our mixed-methods evaluation found that staff and service users experienced positive developments in the move towards service integration through co-location and PIE, with a new culture emerging; yet work on the centre’s vision, goals, and plans for integration was still needed. These findings emphasise the importance of: 1) clear short-, medium-, and long-term plans around the integration of care; and 2) active staff involvement in these plans, to ensure staff investment, effective integration, and service sustainability for PEH.

## Introduction

Homelessness is a growing concern in the UK and internationally.^1,2^ Homelessness is a multifaceted health and social problem,^1^ involving an inequality triad of housing inequality, social inequality, and health inequality, which perpetuates the exclusion of PEH from mainstream services.^3^

Many PEH have complex trauma, multimorbidity, and a high risk of substance misuse, poor mental health, and premature mortality.^4^ Consequently, PEH often have complex and multiple health and social care needs,^5^ requiring targeted approaches.^6^ However, they often face barriers accessing suitable services.^7^ Furthermore, staff working in the area of homelessness face high levels of burnout, post-traumatic stress, and secondary traumatic stress,^8,9^ with high caseloads and inadequate resources, and have highlighted the need for support within the workforce, including strengthening co-working relationships, psychological counselling, and reflective practice.^10,11^

Over the past decade, policies to improve health and social care integration have developed within the nations of the UK. In Scotland, integration of health and social care was legislated in 2014, followed by the establishment of Health and Social Care Partnerships (HSCPs) in 2016. HSCPs consist of healthcare providers and local authorities working together through integrated joint boards to fund and co-develop health and social care services. HSCPs have specific responsibilities to help reduce inequalities and take into account circumstances that may hinder access to services.^12^

Cognisant of the complex needs faced by PEH, the NICE published guidance on integrating health and social care for PEH.^6^ These guidelines underscore the value of service co-delivery (*‘*one-stop shops’), PIE, and trauma-informed care (TIC) training for staff to improve support, access, and service engagement for PEH.^6^ Similar recommendations were expressed in a 2016 review of Edinburgh’s homelessness services by the University of Edinburgh.^13^

Edinburgh’s homelessness services previously operated between two centres across the city: one for housing and social care, and one for health services. Following the 2016 review,^13^ it was recommended to co-locate services under a single-line management structure to enhance joint working and improve service user experience. In November 2021, the two homelessness services moved into one building, the co-located centre (‘the centre’), operating under a single-line management structure. The centre adopted PIE principles (physical environment, psychological framework, staff support and training, managing relationships, and evidence-generating practice)^14^ and TIC as the psychological frameworks to better support PEH.^15^ All staff received level 2 online TIC training by a clinical psychologist before the move, and an on-site clinical psychologist was employed to integrate these principles into the centre’s practices.

The current study was commissioned by the City of Edinburgh Council, who wanted an independent evaluation of the new centre. The study aims were:

- to explore staff views on the expected outcomes of moving the two separate homelessness services into one co-located building, and perceived barriers and facilitators to achieving these; then assess progress of integration and implementation of a PIE 2 years after co-location; and
- to assess service user satisfaction with the co-located centre.

## Method

### Study design

This evaluation was conducted between April 2021 and January 2024, using a mixed-methods approach to assess staff and service user perspectives. For staff, a pre- and post-test design was used, comprising of surveys, interviews, and focus groups before the move (baseline) and 2 years after (follow-up). Service users’ views were collected in a survey 2 years after the move. Participation in the surveys and interviews was voluntary and participants could withdraw at any time.

### Setting

The study took place in Edinburgh, at the separate homelessness services for the baseline evaluation and at the co-located centre for the follow-up evaluation.

### Quantitative methods

#### Staff survey

A baseline online survey was sent to all homelessness services’ staff in Edinburgh between April and May 2021, before TIC training. Participants received an introductory statement outlining the study’s purpose and were assured of the confidentiality and anonymity of their data should they decide to participate. The survey included demographic information, bespoke questions on TIC knowledge, and validated measures of mental health,^16^ stress,^17^ burnout,^18^ team climate,^19^ job satisfaction,^20^ and future work intentions.^21^ A follow-up online survey was sent to all staff at the centre in June 2023, with the same baseline measures and additional bespoke questions on staff support services and perceived service improvements (see Supplementary Information S1). The latter were only answered by staff who had previously worked for Edinburgh’s homelessness services. Data from both surveys were comparatively analysed on IBM SPSS Statistics (version 27), employing parametric (independent *t*-test) and non-parametric tests (Mann–Whitney *U*), as appropriate.

#### Service-user survey

The researchers and staff at the centre, including a staff member with lived experience (JC), developed a bespoke survey, which was conducted from October–December 2023 (see Supplementary Box S2). Using convenience sampling, JC approached 97 service users as they attended the centre. Verbal informed consent was obtained from JC and through an introductory statement prepared by the manager of the co-located centre, which outlined the survey’s purpose and assured confidentiality and anonymity of data. Participants were informed that participation was voluntary and their decision to participate or decline would not affect their care. Participants received a £10 supermarket voucher for participating in the study. The survey included questions on demographics (age, sex, and ethnicity), views on the centre, and two statements from the Scottish Government’s Medication Assisted Treatment (MAT) standards, which aim to ensure consistent and safe drug treatment.^22^ Descriptive analysis was performed on IBM SPSS Statistics (version 27).

### Qualitative methods

Semi-structured individual interviews and focus groups with staff were conducted at baseline and follow-up, focusing on expected outcomes of moving to the co-located centre and barriers and facilitators to achieving these; and the PIE. Follow-up interviews then assessed respective progress after co-location. Staff were sent a participant information sheet before the interviews and verbal consent was obtained at the start of each session after informing participants about the study’s objectives and their right to withdraw at any time. Participants were informed that their identities and any identifiable data would remain confidential and anonymised. Baseline focus groups (*n* = 4) and individual interviews (*n* = 6) were conducted by ED between September and October 2021 (before the move), and 4–8 weeks post-TIC training (see supplementary file for interview guide). A total of 25 staff participated. Focus groups were organised for each professional group within Edinburgh’s homelessness services (housing, social work, health, and administration). Interviews were conducted on video (Microsoft Teams) and telephone owing to COVID-19 restrictions.

Follow-up focus groups (*n* = 5) and individual interviews (*n* = 11) were completed by LN between September 2023 and January 2024, approximately 2 years post-move (see supplementary file for interview guide). A total of 29 staff participated, including 16 baseline participants, with staff from housing, social work, health, administration, and third sector. Focus groups were similarly organised by professional group. Most interviews were held in-person, with four conducted via video (Microsoft Teams).

ED is a social scientist and experienced qualitative researcher who has worked extensively with people with complex needs and health and social care staff who support them over the past 20 years. LN is a GP and university clinical lecturer in general practice with experience in qualitative research. As experienced qualitative researchers, both were mindful of potential power imbalances with participants. Before each interview and focus group, they were transparent about their roles and backgrounds, sharing personal details to foster reciprocity. They ensured participants understood the study’s aims, their rights, and the option to withdraw or skip questions without consequence, while also guaranteeing full anonymity.

All interviews (individual and focus groups) were audio-recorded with oral consent, transcribed verbatim, and analysed using NVivo (version 20). A hybrid deductive and inductive thematic analysis was applied, guided by the thematic framework from baseline findings and new themes from follow-up. Initial coding was completed independently by three researchers (LN, ED, and SWM), with a common coding framework established once consensus was reached. All transcripts were subsequently coded by LN, following Braun and Clarke’s principles.^23^ The phases of thematic analysis, as outlined by Braun and Clarke, were applied at baseline and follow-up in the six following steps: familiarisation with the data; generation of initial codes; searching for themes; reviewing themes; defining and naming themes; and producing the final report. For the baseline qualitative date, two authors (ED and SWM) independently developed initial codes from a set number of interview and focus group transcripts. Individual coding interpretations were presented and fully discussed before final agreement was reached on a final coding framework. For follow-up qualitative data, three authors (ED, LN, and SWM) independently developed initial codes from a set number of interview and focus group transcripts. Individual coding interpretations were presented and fully discussed before final agreement was reached on a final coding framework. The coding framework for the baseline and follow-up interviews is shown in the supplementary file.

## Results

### Staff survey results

The baseline survey achieved a 100% response rate (*n* = 54 participants). The follow-up survey achieved an 89% response rate (*n* = 50 out of 56 staff participating). Staff demographics were consistent across both surveys, with most being female and approximately 7 years of experience (see Supplementary Table S1).

Significant improvements were observed at follow-up in all but one aspect of TIC knowledge, which was just below statistical significance (*P* = 0.057). A significant reduction in staff burnout was also observed, with the proportion of staff experiencing burnout reducing from 74% to 34% at follow-up (*P*<0.001) (Table 1).

**Table 1.** Staff baseline and follow-up scores on the knowledge and delivery of TIC

TIC statements	Baseline score, mean (SD)	Follow-up score, mean (SD)	*P* value
S1: I understand the possible long-term consequences of experiencing complex trauma	4.1 (0.6)	4.6 (0.5)	<0.001
S2: I feel confident at using the concept of the window of tolerance as a tool to understand how someone might present to my service	3.0 (1.1)	4.2 (0.8)	<0.001
S3: This service has adequate policies and practices in place to avoid harming or re-traumatising our clients	2.7 (0.9)	3.2 (1.0)	0.05
S4: My place of work lets me know exactly what I need to do to provide trauma-informed care	2.5 (0.9)	3.6 (0.9)	<0.001
S5: I understand how an experience of trauma might get in the way of someone working or interacting with me effectively	4.2 (0.7)	4.5 (0.6)	0.006
S6: I feel confident in speaking with someone about how their experiences of trauma might be getting in the way of our work together	3.2 (1.0)	3.9 (1.1)	<0.001
S7: I understand the factors which might help someone have a positive experience of disclosing traumatic event	3.4 (0.9)	3.9 (0.8)	0.006
S8: I feel safe talking to my supervisor or manager about how my trauma history impacts my work	2.9 (1.2)	3.5 (1.3)	0.02
S9: I feel safe talking to my supervisor about my experiences of vicarious trauma, compassion fatigue, or burnout	3.1 (1.2)	3.7 (1.2)	0.009
S10: I feel confident I can put some self-care strategies into practice, should I need to	3.5 (1.0)	4.0 (0.9)	0.06
S11: I know how I can access further support in order to look after my wellbeing, should I need to	3.5 (0.9)	4.1 (0.8)	0.002

1 = strongly disagree. 2 = disagree. 3 = neutral. 4 = agree. 5 = strongly agree. *P*-values calculated using Mann–Whitney *U* tests and independent *t*-test. TIC = trauma-informed care.

Changes in the other measures of wellbeing (anxiety and depression) showed a trend towards improvement, except for stress. Team climate showed significant improvements between baseline and follow-up in ‘participation’ (3.0 versus 3.3, respectively; *P* = 0.008), ‘support for innovation’ (3.1 versus 3.3, respectively; *P* = 0.044), and ‘objectives’ (4.2 versus 4.8, respectively; *P* = 0.008). No significant differences in overall job satisfaction were observed (although the mean value was slightly lower at follow-up, indicating an improvement), and job satisfaction over the previous 12 months showed an improvement that was of borderline statistical significance (*P* = 0.051) (Table 2).

**Table 2.** Wellbeing, team climate, job satisfaction, and future intentions of staff

Category	Baseline score	Follow-up score	*P* value
**Wellbeing, mean (SD)**			
Burnout	3.2 (1.3)	2.4 (1.2)	<0.001
Anxiety	2.4 (2.0)	1.9 (1.9)	0.226
Depression	1.9 (2.0)	1.5 (1.9)	0.419
Anxiety and depression	4.2 (3.7)	3.4 (3.7)	0.229
Stress	3.4 (1.2)	3.0 (1.0)	0.152
**Wellbeing caseness, %**			
Anxiety	35	26	0.313
Depression	32	20	0.184
Burnout	74	34	<0.001
Stress	76	80	0.619
**Overall team climate, mean (SD)**			
Participation	3.0 (1.0)	3.3 (0.7)	0.008
Support for innovation	3.1 (0.8)	3.3 (0.8)	0.044
Objectives	4.2 (1.4)	4.8 (1.3)	0.008
Task orientation	4.2 (1.3)	4.5 (1.2)	0.106
**Job satisfaction and future intentions, mean (SD)**			
Overall job satisfaction	3.8 (1.5)	3.4 (1.7)	0.157
Job satisfaction in the past 12 months	2.6 (1.3)	3.0 (1.2)	0.051
Intention to increase hours	1.8 (1.3)	1.6 (1.2)	0.389
Intention to decrease hours	2.5 (1.5)	2.3 (1.4)	0.395
Intention to leave job	3.2 (1.6)	2.9 (1.5)	0.371

Wellbeing measures and caseness: *P*-values calculated using Mann–Whitney *U* tests. Team climate measures: *P*-values calculated using Mann–Whitney *U* tests and independent *t*-test. Job satisfaction and future intentions: *P*-values calculated using Mann–Whitney *U* tests.

The centre’s support services for staff and perceived helpfulness are shown in Table 3. Most staff (88%) had used at least one support service, with the garden, lunch space, and reflective practice being the most commonly utilised. The clinical psychologist’s one-to-one sessions were rated the most helpful, with 53% finding them very helpful.

**Table 3.** Support services for staff at the co-located centre (*N* = 50)

		Was it helpful? *n* (%)		
**Type of support accessed**	**How many accessed the services? *n* (%)**	**Yes, very**	**Yes, a little**	**No**
One-to-one sessions with clinical psychologist	30 (60)	16 (53)	9 (30)	5 (17)
Mindfulness sessions	24 (48)	8 (33)	11 (46)	5 (21)
Reflective practice sessions	36 (72)	14 (39)	16 (44)	6 (17)
Shared lunch space	38 (76)	10 (26)	22 (58)	6 (16)
Garden	42 (84)	21 (50)	17 (40)	4 (10)
Nature walks	8 (16)	2 (25)	4 (50)	2 (25)
I haven’t used any services	6 (12)	—	—	—

**Figure 1.**
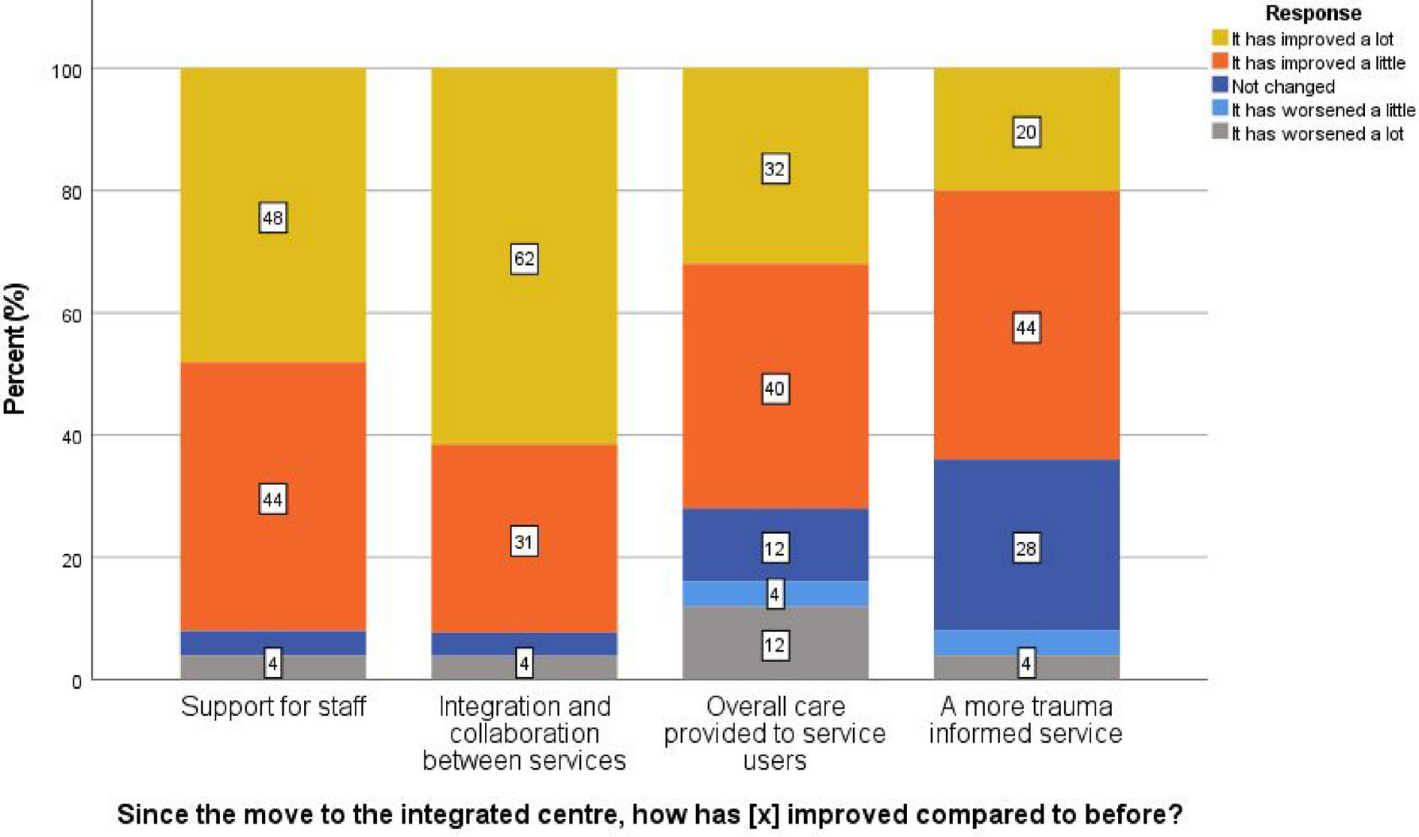
Staff’s perceptions in improvement since moving to the integrated centre

Among staff who worked for the homelessness services before the move to the new centre, 92% felt staff support had improved, 93% reported better integration and collaboration, 64% felt the service was more trauma-informed, and 72% believed the overall care for service users had improved (Figure 1).

### Qualitative findings

**Figure 2.**
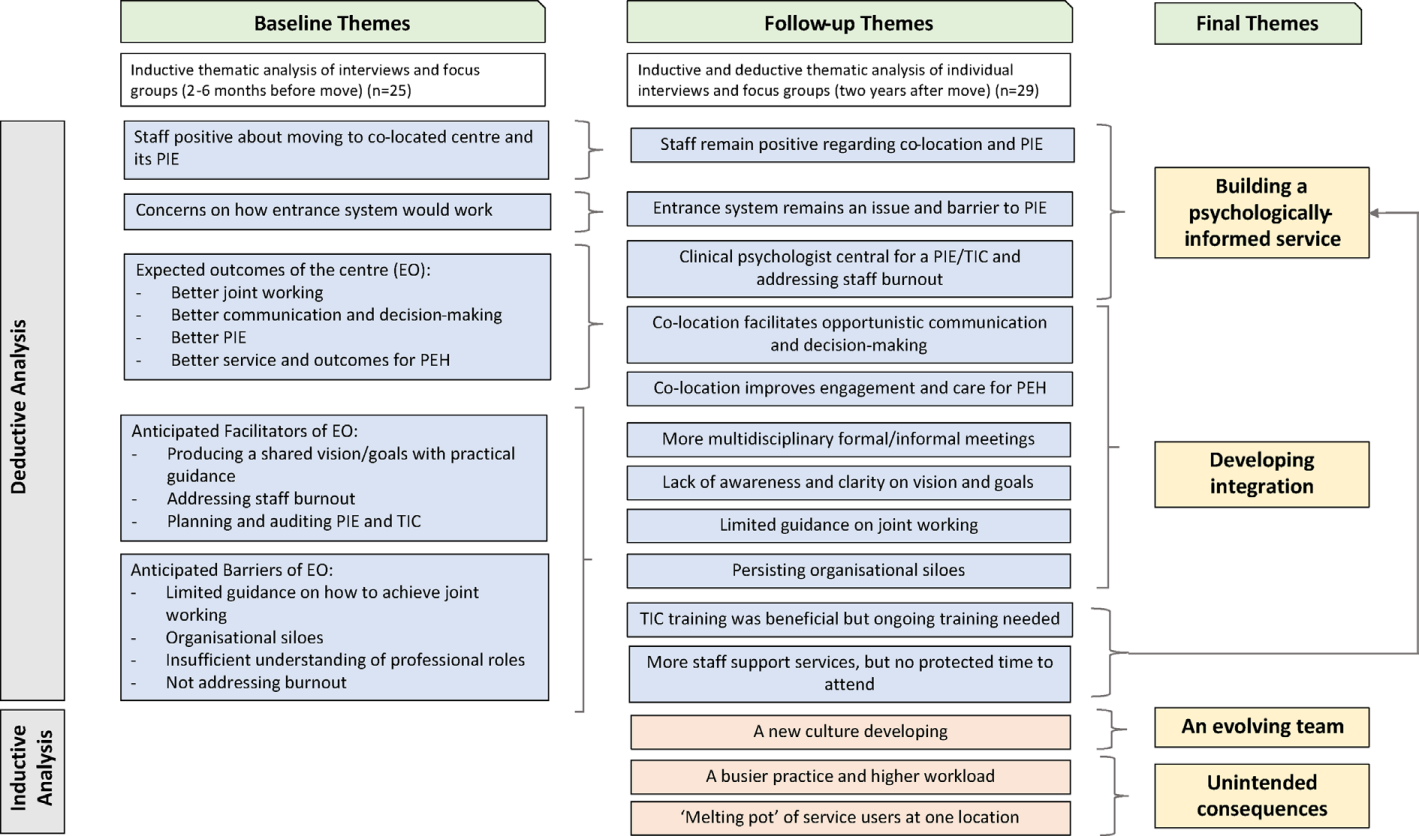
Structure of inductive and deductive thematic analysis applied to staff interviews, with final key themes. EO = expected outcomes. PEH = people experiencing homelessness. PIE = psychologically informed environment. TIC = trauma-informed care.

In the baseline interviews, staff were positive about the co-located centre and its PIE, identifying its expected outcomes and anticipated barriers and facilitators to achieving these. At follow-up, these views were captured in four themes (Figure 2):

1. building a psychologically informed service;
2. developing integration;
3. an evolving team; and
4. unintended consequences.

All quotes below are from follow-up interviews and contextualised with insights from the baseline data.

#### Theme 1: Building a psychologically informed service

TIC within a PIE was a core ethos underpinning the new centre. At baseline, staff anticipated this framework would enhance support for both staff and PEH. Staff reflected on its evolution at follow-up, revealing the following sub-themes.

##### Sub-theme 1: The building

Staff praised the renovated building, describing it as *‘night and day’* (Focus group 3), compared with the previous service buildings. Many were encouraged by the efforts invested in its design, emphasising its positive impact on the care experience:

*‘It’s clean. It’s brightly lit. It suggests we care about you. You matter*.*‘* (Participant 4)

Nevertheless, all interviewees expressed ongoing concerns regarding the entrance system, which restricted entry and exit to one service user at a time and had a malfunctioning intercom:

*‘*[The entrance] *needs attention. There’s far too much hanging about, aggression on the doorstep. It’s quite intimidating. It puts some people off coming here*.*‘* (Focus group 1)

##### Sub-theme 2: The clinical psychologist

In baseline interviews, staff positively anticipated the addition of a clinical psychologist to enhance the centre’s psychologically informed approach, a view that was reinforced at follow-up and commended as essential for a PIE. Additionally, addressing staff burnout was considered a key facilitator to achieving the centre’s expected outcomes at baseline. By follow-up, the psychologist had introduced several staff support services — including one-to-one sessions, mindfulness, and reflective practice — that were widely praised:

*‘In the type of place that we work,* [the psychologist] *is vital for staff, so we don’t burn out, we don’t get to the point that we cannot physically go on any further. That’s one of the best supports*.*‘* (Focus group 3)

The baseline evaluation identified an insufficient understanding of professional roles as a barrier to achieving the centre’s outcomes. At follow-up, the multidisciplinary reflective practice offered by the psychologist was seen as a way to mitigate this, fostering better understanding and cohesion among the different professions:

*‘It’s a new learning and realisation, that actually another service or team within the service may be struggling with that. That helps people develop a sense of connection with each other. They remember ... the cases that are brought, the similarities, the shared values*.*‘* (Participant 4)

However, accessing support was often challenging owing to high workloads and a lack of protected time for staff to attend.

##### Sub-theme 3: Trauma-informed care

In baseline interviews, embedding TIC was anticipated as a key facilitator to achieving the centre’s expected outcomes. At follow-up, most confirmed that TIC training was beneficial and led to a shared understanding of service users’ needs:

*‘It’s given people a way to see human experiences differently … It’s more compassionate*.*‘* (Participant 7)

Many staff acknowledged the psychologist’s key role in informing the centre’s trauma-informed approach and emphasised the need for ongoing training.

##### Sub-theme 4: Better services for PEH

In baseline interviews, a key expected outcome for the centre was a better service for PEH. At follow-up, co-located services were seen as invaluable for enhancing engagement and care for this group:

*‘Being able to get a range of needs met under one roof … that’s a massive difference*.*‘* (Participant 4)

Staff noted that previously, service users struggled to attend multiple appointments across town, limiting possibilities for holistic care.

### Theme 2: Developing integration

#### Sub-theme 1: Progress from key expected outcomes

Alongside a better PIE, expected outcomes from the baseline interviews included improved communication, decision making, and joint working. At follow-up, some staff highlighted that co-location enhanced their understanding of each other’s roles:

*‘We’re learning about it all. I’m learning about doctors, community psychiatric nurses, social work, housing. We have all these chats with all the different people from different departments. It helps staff grow and be more educated and have more knowledge and wisdom of what goes on*.*‘* (Participant 6)

Co-location enabled opportunistic communication, which streamlined decision making and led to more timely and efficient care for service users, when executed effectively:

*‘I saw the benefit from day one. The speed with which I could get information in order to inform my decision making improved no end. The ability to then increase and build upon the personal, professional relationships to facilitate joint working*.*‘* (Participant 2)

However, integration at system level was felt to be lacking:

*‘As much as we’re trying to be an integrated service, there is still progress to be made. Sometimes people will come in for housing but they’re due X from health, and health may have just missed that they’re in the building*.*‘* (Participant 3)

Some staff highlighted the impact of informal communication on their work, with one professional noting *‘sometimes people feel a bit put upon’* (Participant 4) when interrupted.

Furthermore, staff reported ongoing organisational siloes, which was previously identified as a barrier to achieving the centre’s outcomes. With a larger team, staff emphasised the need for cohesive and intentional joint-working practices to facilitate integration:

*‘Because homelessness is split between council, NHS, third sector organisations, charities … It’s very easy for everyone to go off and do their own thing, and any cohesion can get lost. You have to actively be aware of* [this] *and make the effort to speak to people*.*‘* (Focus group 5)

Another issue was the lack of unified eligibility criteria for service users. The different criteria across professional groups created challenges for integrating care and led to service-user frustrations:

*‘It does feel a bit like three, plus the voluntary sector, services all sharing the same place, rather than a fully integrated service. My view would be, if you come into the building, you can get everything if needed ... We’ve come with our own criteria, but we haven’t managed to knock it together and say, "Here’s when it starts for everyone". That can be frustrating for the service users, that they only get some of the service*.*‘* (Focus group 1)

Furthermore, the absence of an integrated IT system and shared service-user data posed a considerable barrier to service integration.

#### Sub-theme 2: Multidisciplinary gatherings

At baseline, an insufficient understanding of different professional roles was identified as a barrier to achieving the centre’s outcomes. To address this, the centre instituted formal multidisciplinary meetings, including management team meetings, away days, interdisciplinary case reviews, and whole team meetings.

Management team meetings, involving the single-line manager and lead managers from each professional group, allowed managers to share insights and *‘disseminate learning more reflectively’* (Participant 8) to their teams.

Additionally, case reviews were particularly valued for joint working, as it enabled staff to discuss complex service users with multidisciplinary feedback:

*‘We spent months banging our heads off the wall, then bringing everybody together* [at the case review] *... That was really helpful*.*‘* (Focus group 1)

Notably, informal gatherings were regarded as central to creating an integrated ethos:

*‘A lunch room, kitchen, and garden space that we can all share, and spend time, not talking about work. Having the quick conversation at the coffee machine. That makes a big difference as well*.*‘* (Focus group 3)

Additionally, interdisciplinary desk mixing was seen as important for promoting integration, as it encouraged opportunistic information sharing and a greater understanding of each other’s roles. However, not all staff utilised these spaces, leading to persisting siloes.

#### Sub-theme 3: More clarity on the centre’s vision and goals

At baseline interviews, staff identified a shared vision and goals as a key facilitator to achieve the centre’s expected outcomes, alongside a practical roadmap for implementation. Correspondingly, limited guidance was seen as a barrier. At follow-up, most expressed that the shared vision and roadmap were still not fully established:

**Responder 1:** *‘I don’t remember the roadmap layout, the first time round*.*‘*

**Responder 2:** *‘I’ve never been told about a roadmap*.*‘*

**Responder 3:** *‘Neither have I*.*‘*

**Responder 4** ***:** ’It was something* [the manager] *continued to bring up, over a year and a half ago … because we’re so fire-fighting mode, it’s hard to work towards those*.*‘* (Focus group 3)

Several staff highlighted the need for clearer and more manageable goals:

*‘It does need to be tidied up, made more manageable and very carefully thought-out*.*‘* (Participant 10)

Finally, most were unaware of future plans to facilitate integration and expressed uncertainty over the next steps, expressing concerns about potential budget cuts:

*‘We are three services working in the same building. I would hope we could become more integrated and ultimately more accessible as a service without the burden to staff … I worry we’re going to have to cut as opposed to increase resource, which is what we want to do*.*‘* (Participant 11)

Overall, staff acknowledged that the centre’s integrated frameworks necessitated further work, reiterating the importance of practical guidance to achieve effective integration:

*‘We haven’t quite got some of the core stuff right. I worry we have tried to do too much, too quickly. Because the expectation is that we would ... It might have been better to say "Yes, we can do those things, but let’s have a timeline*".’ (Participant 1)

*‘It would be good if the vision wasn’t just "We’re going to be more integrated", but here’s what that means in big terms*.*‘* (Focus group 1)

### Theme 3: An evolving team

A notable theme that emerged at follow-up was the emergence of a new culture, one embracing integration and PIE to best support PEH and staff:

*‘There’s a sense of a mission about working here. It’s a personal thing, as well as a corporate slogan. The trauma training creates an identity for the centre*.*‘* (Focus group 1)

The management team was praised for their supportive approach and modelling this team identity:

*‘The support from managers makes a big difference to how you can provide a service. Everybody here comes from wanting to provide a good service to the people we support*.*‘* (Participant 6)

However, there was recognition that some staff were struggling to adapt:

*‘It comes along with the challenges of cultural change. Not everyone’s ready. There are people who are early adopters and there’s others who aren’t really interested*.*‘* (Participant 4)

### Theme 4: Unintended consequences

Despite the benefits of co-location, moving two services into one building brought its own set of challenges, notably greater footfall from PEH.

#### Sub-theme 1: A busier practice

Co-location led to a busier practice with considerably higher numbers of service users and a workload that outmatched supply:

*‘We’ve taken two really busy services, put them together, and now we’re uber busy*.*‘* (Focus group 3)

This increased demand resulted in limited consultation spaces and long wait times, causing frustration among service users. The heavy workload also hindered efforts to improve integration and access staff support:

*‘That’s the main focus of everybody, when we’re here … They don’t think beyond the fact that we’re limited in staffing and resources*.*‘* (Focus group 5)

This was further exacerbated by a lack of external resources and service cuts, limiting the provision of quality care:

*‘We are trying to provide integrated services to a large number of people. It seems to be growing, but the resource doesn’t necessarily seem to be changing to match it*.*‘* (Participant 1)

#### Sub-theme 2: A ‘melting pot’ of service users

The other unanticipated outcome was the range of service users, with different needs and vulnerabilities, attending one location, with one interviewee describing it as *‘a melting pot’* (Participant 5):

*‘The amount of incidents that’s happened in this building, since we’ve all been together, is pretty outrageous. Probably on a daily basis, something happens with our patients. Either someone gets screamed at, maybe arguing in the waiting room with each other … It can be a hotspot. The tensions, stimulus is too high*.*‘* (Focus group 3)

A newly implemented strategy, heralded by staff, was the presence of an administrative member in the waiting area:

*‘It de-escalates situations, or prevents them from happening before de-escalation is required. Service users feel more welcome, more secure, more able to ask for more things.* [The staff] *can unpack things in a space that’s not rushed. That is vital for integration*.*‘* (Focus group 3)

Staff also discussed that some PEH felt unsafe attending the centre, owing to fears of encountering an adversary or concerns related to past trauma and not feeling safe in this environment:

*‘Clients’ own relationships amongst themselves can cause issues in reception. There’s a lot of debt, or purchasing gone wrong*.*‘* (Focus group 2)

### Service-user survey results

The survey achieved an 86% response rate (*n* = 83 out of 97). Responder characteristics are detailed in Supplementary Table S2, with no significant differences in responses across demographics.

Satisfaction with the centre was high, with 72% satisfied with the overall service, 75% finding the services easier to access, and 78% happy with the services provided (Table 4). Furthermore, 78% felt safe within the building and 75% expressed confidence in the service (see Supplementary Figure S1).

**Table 4.** Service users’ perceptions on the integrated centre (*N* = 83)

**Category**	Service user responses
**Overall satisfaction with integrated centre*, n* (%)**	
Poor	7 (8)
Fair	16 (19)
Good	24 (29)
Very good	19 (23)
Excellent	17 (20)
**Easier to access services compared with before, *n* (%)**	
Does not apply	17 (20)
Much harder	1 (1)
No different	3 (4)
Slightly easier	4 (5)
Much easier	58 (70)
**Satisfaction with the services at the centre, *n* (%)**	
Very dissatisfied	1 (1)
Quite dissatisfied	7 (8)
Neutral	10 (12)
Quite satisfied	30 (36)
Very satisfied	35 (42)

## Discussion

### Summary

Two years after co-location to the new centre for PEH, statistically significant improvements were observed in staffs’ knowledge of TIC, burnout levels, and most team climate domains. Improvement in staff job satisfaction over the previous 12 months was of borderline statistical significance (*P* = 0.051). The new staff support services were well-used and helpful. Staff, who worked for the services previously, also reported better support, service integration, TIC practice, and service-user care at the co-located centre. Service users also expressed satisfaction, feelings of safety and confidence, and increased accessibility with the centre’s services.

Interviews showed positive developments towards service integration and PIE, with the emergence of a new culture. However, system-level integration still faced challenges, including persisting organisational siloes and a lack of clarity on the short- and long-term plans of integration. Unforeseen challenges included a busier practice, higher staff workloads, and managing the increased complexity of service users in one location.

### Strengths and limitations

Strengths include the mixed-methods design, high survey response rates, and staff and service-user involvement. The service-user characteristics are broadly comparable with PEH characteristics across Scotland.^24^ The use of validated measures in the staff surveys was a strength, although the use of single-item questions to assess staff job satisfaction may have been a limitation, as job satisfaction is a complex construct, influenced by a great many factors, and is probably best measured by a range of items. This was done for pragmatic reasons to limit the length of the survey but may have been a reason why overall job satisfaction did not improve significantly.

Other limitations include the fact that the baseline evaluation was conducted during the COVID-19 pandemic, which may have influenced staff wellbeing and job satisfaction results, although there is evidence to refute this assertion.^25,26^ The evaluation period was relatively brief, limiting insights into long-term sustainability and the full impact of integration.

Convenience sampling in the service-user survey may have introduced selection bias, and the financial incentive may have influenced participants to give more favourable responses about the services. Thus, we cannot be sure that the service-user survey findings are representative of service users using the co-located homelessness centre more generally. Finally, the study’s focus on one co-located homelessness service may limit the generalisability of the results; still, it may offer greater real-world applicability for integrated service models in similar contexts.^27^

### Comparison with existing literature

To our knowledge, this is the first study evaluating a co-located homelessness centre in the UK and wider Europe, with most research previously conducted in North America.^28–30^

Both our present research and previous studies indicate that co-location can support integrated service delivery by fostering formal and informal integration through shared spaces.^28,31^ Research on co-located homelessness services in the US has shown improved access to care, better engagement with primary and preventive services, higher service-user satisfaction, and reduced emergency visits among PEH.^28^ However, establishing effective joint-working frameworks is essential to translate the benefits of co-location into fully integrated care and better outcomes.^31^

Our study also echoes previously identified barriers to service integration, including enduring organisational siloes, inadequate communication channels, a lack of clear vision and goals, high workloads, and poor technological integration between professional groups.^32,33^

### Implications for research and practice

Previous studies indicate that TIC and PIE are feasible interventions with overall positive benefits on both staff and PEH.^5,34–36^ Our study contributes to this literature by underscoring the importance of psychologically informed physical environments in enhancing the care experience, corroborating findings on trauma-informed design in homeless shelters.^37^ Moreover, our evaluation highlights the value of an on-site clinical psychologist to facilitate a PIE by supporting staff and promoting psychologically informed perspectives, similar to a recent evaluation of trainee clinical psychologists in homelessness settings.^38^ Given the high levels of traumatic stress, burnout, and staff turnover faced within the homelessness workforce,^8–10^ our findings suggest that incorporating clinical psychologists within homelessness services is highly advantageous.

A key finding from our evaluation was the need for a clear vision and defined goals to facilitate better joint-working and service integration. Effective integration in integrated care models relies on a shared and regularly communicated vision and goals to enhance staff commitment and sustainability.^32,33,39^ When the vision and goals are not representative of all staff, it may significantly hinder integration progress.^37^

In conclusion, effective integrated care models require clear long-term plans, with dedicated, ring-fenced funding and resources to ensure sustainable integration,^32,33^ often taking several years before significant changes are observed.^39^ These challenges are further amplified for PEH, with limited resources allocated to adequately address integrated care.^1^ While our evaluation highlights positive strides towards integration and PIE 2 years on, the importance of a clear vision and well-defined goals outlining short-, medium-, and long-term integration plans, with regular staff involvement, collaborative leadership, and adequate funding, are crucial to build on the benefits of a co-located service.

## Data

The authors do not have ethical approval or patient consent to share the full dataset.
